# Synchronous Prostate Adenocarcinoma and Bladder Carcinoma In Situ Detected During Evaluation of Incidental PSA Elevation: A Case Report Illustrating Multimodal Diagnostic Correlation and Long-Term Follow-Up

**DOI:** 10.3390/reports9020166

**Published:** 2026-05-22

**Authors:** Simona Maria Borta, Adrian Silviu Crișan, Imola Miklos, Dana Zdremtan, Roxana Andra Coman

**Affiliations:** 1 Department of Internal Medicine, Faculty of Medicine, “Vasile Goldiș” Western University from Arad, B-dul Revoluției Nr. 94-96, 310025 Arad, Romania; simoborta@yahoo.com; 2 Department of Intensive Care and Emergency Medicine, Faculty of Medicine,“Vasile Goldiș” Western University from Arad, B-dul Revolutiei Nr. 94-96, 310025 Arad, Romania; adriancrisan74@yahoo.com; 3 Department of Biology and Life Science, Faculty of Medicine, “Vasile Goldiș” Western University from Arad, B-dul Revoluției Nr. 94-96, 310025 Arad, Romania; 4 Department of Otorynolaryngology, “Vasile Goldiș” Western University from Arad, B-dul Revolutiei Nr. 94-96, 310025 Arad, Romania; zdremtan.dana@uvvg.ro; 5 Department of Biochemistry, Faculty of Medicine, “Vasile Goldiș” Western University from Arad, B-dul Revolutiei Nr. 94-96, 310025 Arad, Romania; 6 Department of Urology, MedLife Humanitas Hospital, 400664 Cluj-Napoca, Romania; dr.roxanacoman@yahoo.com

**Keywords:** prostate cancer, bladder carcinoma in situ, SelectMDx, PSA, multiparametric MRI, synchronous tumors, neutrophil-to-lymphocyte ratio, biomarker integration

## Abstract

**Background and Clinical Significance**: The coexistence of synchronous urologic malignancies may present diagnostic and therapeutic challenges, particularly when symptoms are minimal or nonspecific. This case illustrates the role of multimodal diagnostic correlation in identifying a second primary urologic malignancy during the evaluation of incidental PSA elevation. Case presentation: **Case Presentation**: We report the case of a 56-year-old male presenting with minimal lower urinary tract symptoms who underwent stepwise diagnostic evaluation including PSA (prostate specific antigen), free PSA, urinary SelectMDx RT-PCR testing (reverse transcription polymerase chain reaction), multiparametric MRI (magnetic resonance imaging), transrectal biopsy and inflammatory biomarker assessment. PSA was 17.69 ng/mL with a free PSA ratio of 6.56%. SelectMDx indicated a 90% probability of prostate cancer and a 65% risk of Gleason ≥ 7 disease. mpMRI demonstrated two suspicious lesions without extracapsular extension. Biopsy confirmed acinar adenocarcinoma Gleason 7 (3 + 4), Grade Group 2. Persistent post-biopsy hematuria led to additional imaging that revealed bladder wall thickening, and cystoscopy confirmed multifocal carcinoma in situ. Radical cystoprostatectomy with orthotopic ileal neobladder reconstruction was performed. **Conclusions**: This case illustrates the importance of diagnostic vigilance and multimodal correlation in a minimally symptomatic patient, particularly when persistent clinical findings are not fully explained by the initial diagnosis. The findings should be interpreted as illustrative and cannot be generalized beyond the single-case context.

## 1. Introduction and Clinical Significance

Synchronous urologic malignancies, particularly prostate adenocarcinoma and bladder carcinoma in situ (CIS), represent a diagnostic challenge due to overlapping symptomatology and shared risk factors [1,2]. While prostate specific antigen (PSA) remains the most accessible biomarker for prostate cancer detection, its limited disease specificity has prompted the development of adjunct molecular assays [3]. Urinary-based gene expression tests such as SelectMDx have demonstrated improved risk stratification for clinically significant prostate cancer (Gleason ≥ 7) and may reduce unnecessary biopsies [4,5].

Multiparametric magnetic resonance imaging (mpMRI) has become a central component of prostate cancer diagnostics, improving lesion detection and guiding targeted biopsy strategies [6,7]. When combined with molecular assays, imaging may contribute to the predictive accuracy and diagnostic concordance [8].

The coexistence of multiple primary urologic malignancies represents a clinically challenging scenario requiring careful diagnostic evaluation. Although prostate cancer and bladder cancer share several risk factors, including age, smoking history and environmental exposures, synchronous presentation remains less commonly identified in routine diagnostic pathways [9,10,11].

Reported incidence rates of synchronous prostate cancer and bladder carcinoma in situ vary depending on the studied population and diagnostic context, with estimates generally ranging between 1% and 6% in clinically detected cases [12,13].

However, higher rates of incidental prostate cancer have been reported in radical cystoprostatectomy specimens, often reaching 20–50% in surgical series, reflecting differences in detection methods and population characteristics [14,15].

These observations suggest that the apparent rarity of synchronous disease may be influenced by diagnostic pathways rather than true biological absence.

In clinical practice, the detection of one malignancy may overshadow the presence of another, particularly when symptoms are minimal or nonspecific. Consequently, comprehensive diagnostic assessment remains essential in patients presenting with atypical clinical findings or persistent unexplained hematuria [16]. In this context, multimodal diagnostic strategies integrating serum biomarkers, urinary molecular assays and advanced imaging techniques may provide complementary information in complex diagnostic scenarios [17,18].

Synchronous urologic malignancies may follow different diagnostic pathways, potentially delaying recognition of a second primary tumor. Persistent or discordant clinical findings may therefore justify broader multimodal urothelial evaluation [12,16,17,18].

We present a case illustrating multimodal diagnostic correlation between serum PSA, urinary molecular testing, imaging findings, histopathology and systemic inflammatory status in a patient with synchronous prostate adenocarcinoma and bladder carcinoma in situ.

Clinical Significance: this case illustrates the importance of continued diagnostic evaluation when persistent clinical findings are not fully explained by the initial diagnosis. The report also provides six-year follow-up after radical cystoprostatectomy with orthotopic ileal neobladder reconstruction, without evidence of oncological recurrence.

## 2. Case Presentation

### 2.1. Patient Characteristics and Initial Evaluation

In 2019 a 56-year-old urban male patient presented with minimal urinary discomfort. Past medical history included grade I arterial hypertension under medical control. The patient was a former smoker (cessation 15 years prior), with no occupational toxic exposure and no significant oncologic family history.

Initial laboratory testing (performed at Synevo Romania) revealed:

- Total PSA: 17.69 ng/mL
- Free PSA ratio: 6.56%

Given the markedly elevated PSA level and unfavorable free PSA ratio, prostate biopsy was clinically indicated. Urinary SelectMDx (MDxHealth B.V., Nijmegen, The Netherlands) testing was additionally performed as an adjunctive molecular assessment to further characterize the risk of clinically significant disease.

### 2.2. Molecular and Imaging Assessment

Urinary SelectMDx (RT-PCR) testing demonstrated:

- 90% probability of prostate cancer detection
- 65% probability of Gleason score ≥ 7 disease

**Figure 1:**
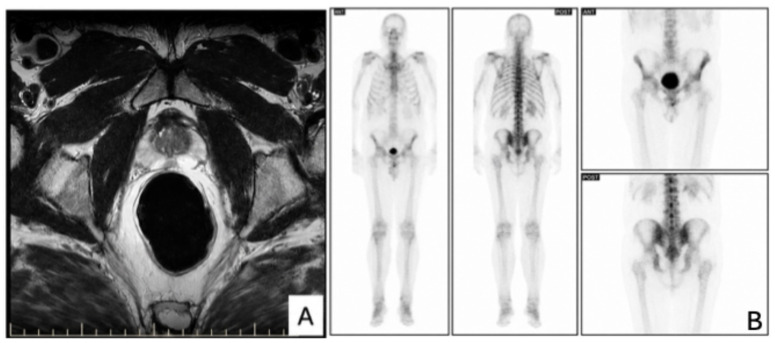
Preoperative imaging evaluation. (**A**) Axial T2-weighted multiparametric MRI of the prostate demonstrating a focal hypointense lesion in the peripheral zone, suspicious for clinically significant prostate cancer (PI-RADS 4–5). Diffusion-weighted imaging (DWI) with corresponding apparent diffusion coefficient (ADC) reduction supported restricted diffusion. (**B**) Whole-body bone scintigraphy showing no evidence of skeletal metastatic disease.

Multiparametric MRI (performed at Affidea, device/tracer details unavailable) (Figure 1A) of the prostate identified two suspicious nodules (PI-RADS 4–5):

- Left apical lesion
- Right apical-medioprostatic lesion

No extracapsular extension, seminal vesicle invasion or pelvic lymphadenopathy was observed.

No bladder wall abnormality was identified on the preoperative mpMRI (performed at Affidea, device/tracer details unavailable); however, the examination was primarily performed and interpreted for prostate evaluation. VI-RADS assessment was not applied at that stage.

Whole-body bone scintigraphy was negative for metastatic disease (Figure 1B) (performed at Timișoara County Emergency Clinical Hospital; device/tracer details unavailable).

Clinical staging was cT2cN0M0.

### 2.3. Histopathological Confirmation

Transrectal prostate biopsy confirmed:

- Acinar adenocarcinoma
- Gleason score 7 (3 + 4)
- Grade Group 2

Pathological staging after surgery later confirmed pT2cN0M0 disease.

Histopathological diagnosis was based on standard hematoxylin–eosin (H&E)-stained sections; reagent manufacturer details were not available in the reviewed documentation.

### 2.4. Detection of Synchronous Bladder Carcinoma

Persistent hematuria developed several days after the prostate biopsy rather than immediately post-procedurally. The bleeding was macroscopic, intermittently associated with blood clots, and did not resolve under empirical antibiotic therapy.

Although post-biopsy hematuria is common, it is typically self-limiting and occurs shortly after the procedure. In this case, the delayed onset, persistence, clot formation, and lack of response to conservative management raised concern for an alternative or additional source of bleeding, prompting further urothelial evaluation.

**Figure 2:**
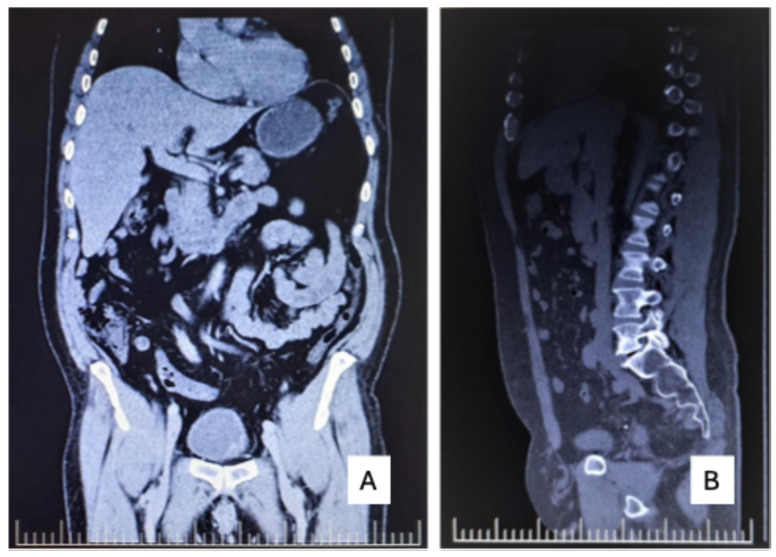
Contrast-enhanced Uro-CT demonstrating bladder wall abnormalities. (**A**) Coronal contrast-enhanced CT reconstruction showing focal thickening and enhancement of the left bladder wall, suspicious for urothelial pathology. (**B**) Sagittal reconstruction confirming focal bladder wall thickening. A non-obstructive right iliac ureteral calculus is additionally noted.

Uro-CT (performed at Affidea, device/tracer details unavailable) revealed left bladder wall thickening (Figure 2A) and a non-obstructive right iliac ureteral calculus (Figure 2B).

Urinary cytology demonstrated numerous erythrocytes (both intact and lysed), superficial and intermediate urothelial cells, and the presence of atypical urothelial cells characterized by enlarged, hyperchromatic and pleomorphic nuclei with irregular nuclear contours. These cytological findings were highly suspicious for high-grade urothelial neoplasia.

Subsequent cystoscopy with transurethral resection confirmed multifocal high-grade urothelial carcinoma in situ (pTisNxM0). No papillary high-grade component was identified.

### 2.5. Treatment Decision and Rationale

An important aspect requiring clarification is the choice of upfront radical cystoprostatectomy instead of intravesical Bacillus Calmette–Guérin (BCG) therapy for bladder carcinoma in situ.

Although BCG represents the standard first-line treatment for high-risk non-muscle-invasive bladder cancer, including CIS, the present case demonstrated extensive, multifocal high-grade CIS involving a large proportion of the bladder mucosa, suggesting a diffuse urothelial field change. Such patterns are associated with increased risks of recurrence and progression and may show suboptimal response to intravesical therapy.

The patient was informed about bladder-preserving strategies, including intravesical BCG therapy, versus radical surgical management, and opted for a definitive surgical approach. Long-term adherence to intravesical treatment protocols and surveillance requirements was also considered in the decision-making process.

It is also important to acknowledge that, in real-world settings, the availability and reimbursement of BCG therapy in Eastern European healthcare systems, including Romania, may be variable. At the time of diagnosis, access to BCG was not uniformly guaranteed, with potential limitations related to institutional availability, supply constraints, and reimbursement policies.

Therefore, the decision for upfront radical cystoprostatectomy was not based on formal contraindications to BCG therapy, but rather on the extent and multifocality of CIS, the coexistence of a second primary malignancy, patient preference, and the feasibility of reliably delivering a complete intravesical treatment course. This approach is consistent with a risk-adapted strategy in selected high-risk cases, where early radical surgery may be considered when multiple unfavorable factors coexist (Table 1).

In the present case, the coexistence of extensive multifocal high-grade CIS and clinically significant prostate adenocarcinoma, together with considerations related to treatment feasibility and adherence, supported the selection of upfront radical cystoprostatectomy as the most appropriate definitive oncological strategy.

### 2.6. Surgical Management and Outcome

After multidisciplinary tumor board evaluation, radical laparoscopic cystoprostatectomy with bilateral lymphadenectomy and intracorporeal orthotopic ileal neobladder reconstruction was performed. Given the coexistence of clinically significant prostate adenocarcinoma and multifocal high-grade urothelial carcinoma in situ, radical cystoprostatectomy was considered the most appropriate definitive oncological strategy, allowing simultaneous management of both malignancies in a single surgical procedure.

The early postoperative course was monitored through routine laboratory parameters, imaging assessment and microbiological surveillance of urinary cultures. Particular attention was paid to infectious complications, as urinary diversion procedures are associated with an increased risk of bacterial colonization during the early adaptation phase of the neobladder. Postoperative renal function remained stable, and imaging studies confirmed appropriate urinary drainage without evidence of obstruction or urinary leakage. These perioperative observations provided additional confirmation of the technical success of the reconstructive procedure and supported the favorable early oncological outcome observed in this patient.

Final histopathology exam confirmed:

- Acinar prostate adenocarcinoma, Gleason 7 (3 + 4), Grade Group 2
- Multifocal urothelial carcinoma in situ pTisN0M0
- Bilateral pelvic lymphadenectomy was performed. All submitted lymph nodes were negative for metastatic involvement (pN0); however, the exact number of retrieved lymph nodes was not available in the reviewed documentation.

Postoperative evolution was complicated by:

- Recurrent urinary tract infections (*Proteus* spp.)
- Acute left pyelonephritis (*Klebsiella* spp.)
- Superinfected pre-existing cortical renal cyst.

**Figure 3:**
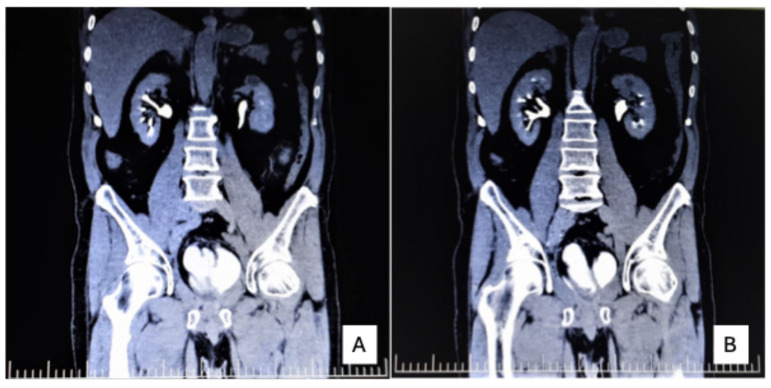
Contrast-enhanced Uro-CT demonstrating postoperative findings and inflammatory complications. (**A**) Coronal contrast-enhanced CT reconstruction showing focal hypodense areas in the left kidney, consistent with acute pyelonephritis, along with a cortical renal cyst. (**B**) Coronal reconstruction demonstrating the orthotopic ileal neobladder within the pelvic cavity, with normal postoperative appearance.

CT imaging showed hypodense areas consistent with acute pyelonephritis and a cortical cyst (Figure 3A,B). Under antibiotic therapy, inflammatory markers normalized, and urine cultures became negative.

Daytime continence was regained at 3 months; nocturnal incontinence improved but persisted partially. Erectile dysfunction was permanent due to oncologic nerve sacrifice.

### 2.7. Systemic Inflammatory Status

Inflammatory markers, including erythrocyte sedimentation rate (ESR = 10 mm/h), C-reactive protein (CRP < 6 mg/L), fibrinogen (235 mg/dL), and the calculated neutrophil-to-lymphocyte ratio (NLR = 1.8) (performed at Policlinica AS), were within normal reference ranges. No metastatic progression was observed. While causality cannot be inferred, the low systemic inflammatory burden was compatible with the absence of advanced disease in this patient.

From a clinical standpoint, these inflammatory parameters were interpreted as supportive rather than decisive findings. They did not establish the diagnosis, but they contributed to the overall impression of limited systemic tumor burden in the absence of metastatic disease or severe ongoing inflammatory activation at that stage. Their inclusion in the present case is relevant because they complement the biomarker-based narrative of the report and illustrate how laboratory context may refine, even if not determine, the interpretation of imaging and histopathological findings.

Although inflammatory biomarkers alone cannot be used for oncological staging, their integration with imaging and histopathological findings may provide additional clinical context when evaluating the overall disease status of patients with urologic malignancies.

### 2.8. Long-Term Surveillance and Late Hematuria Episode

Long-term follow-up in patients undergoing radical cystoprostatectomy with orthotopic urinary diversion requires a multidisciplinary surveillance strategy that includes clinical examination, laboratory investigations and periodic imaging assessment. In addition to oncological monitoring, particular attention must be given to potential functional complications associated with ileal neobladder reconstruction, including metabolic alterations, urinary infections and continence status.

In the present case, follow-up evaluations included periodic PSA monitoring, imaging studies and urological assessment. No biochemical evidence of prostate cancer recurrence was detected during the surveillance period. Renal function remained stable, and no structural abnormalities of the upper urinary tract were identified on follow-up imaging. The overall clinical course therefore reflected both satisfactory oncological control and acceptable functional adaptation to the urinary diversion.

The patient remained recurrence-free from 2019 until autumn 2025, with regular oncologic follow-up.

In autumn 2025, he presented with a new episode of macroscopic hematuria. Urinary cytology revealed inflammatory background with erythrocytes and urothelial cells showing mild to moderate dysplasia without definitive high-grade atypia.

Considering the presence of an orthotopic ileal neobladder, the cytological findings were interpreted cautiously, as reactive and metaplastic epithelial changes may occur in intestinal urinary diversions and can mimic dysplastic alterations.

Given the persistence of hematuria, the history of synchronous malignancies, and equivocal cytological findings in the context of an orthotopic ileal neobladder, further evaluation was considered necessary. Uro-CT and cystoscopy did not reveal any evidence of tumor recurrence.

Although PET-CT (performed at Centrul PET/CT Pozitron-Diagnosztika Oradea) is not routinely used in the surveillance of bladder carcinoma in situ or intermediate-risk prostate cancer, it was performed in this case as a problem-solving imaging modality to exclude occult local recurrence or distant metastatic disease when conventional imaging and cystoscopic evaluation were inconclusive.

Whole-body PET-CT showed no evidence of local recurrence, upper urinary tract dissemination, or distant metastases.

The patient remains under active surveillance.

Follow-up included PSA monitoring every 3 months during the first year, every 6 months thereafter, and annually after 3 years. Imaging (CT thorax–abdomen–pelvis) was performed periodically or when clinically indicated. PET-CT was used selectively as a problem-solving modality in the setting of inconclusive conventional imaging or atypical clinical findings (Table 2).

### 2.9. Chronological Diagnostic and Therapeutic Timeline

To facilitate a clearer overview of the diagnostic pathway, therapeutic interventions and long-term follow-up of this patient, the main clinical events are summarized chronologically. The timeline highlights the sequence of laboratory investigations, molecular testing, imaging findings, histopathological confirmation, surgical management and subsequent surveillance, illustrating the integrated multimodal diagnostic approach applied in this case (Table 3).

The chronological representation of the diagnostic and therapeutic pathway highlights the complexity of managing patients with synchronous urologic malignancies. In this case, the integration of laboratory biomarkers, molecular assays, imaging modalities and histopathological confirmation allowed for a comprehensive evaluation of disease status at multiple stages of the clinical course.

Such structured timelines may be particularly useful in complex oncological cases because they provide a clear overview of the sequence of diagnostic decisions and therapeutic interventions. In addition to improving clarity for clinical reporting, they may also facilitate interdisciplinary communication between urologists, radiologists, oncologists and pathologists involved in patient management. The present timeline therefore illustrates not only the evolution of the disease but also the role of coordinated multimodal diagnostics in guiding clinical decision-making.

### 2.10. Patient Perspective

The patient reported that the initial diagnosis of prostate cancer was unexpected, as symptoms were minimal and nonspecific. The subsequent identification of a second malignancy was perceived as particularly distressing.

Following surgery, the patient described a gradual adaptation to the orthotopic ileal neobladder, with satisfactory daytime continence achieved within a few months, although some degree of nocturnal incontinence persisted.

The patient emphasized the psychological impact of long-term oncological surveillance, particularly during the episode of hematuria in 2025, which raised concerns regarding recurrence. However, the absence of disease progression during follow-up was perceived as reassuring. Overall, the patient expressed satisfaction with the treatment outcome despite the complexity of the clinical course.

## 3. Discussion

This case illustrates a high level of diagnostic concordance between serum PSA, free PSA ratio, urinary molecular profiling, mpMRI and histopathological findings, while also highlighting the limitations of interpreting concordance from a single-patient observation. However, each diagnostic modality has inherent limitations that must be critically appraised. PSA remains organ-specific but not disease-specific, and elevated levels may occur in benign prostatic hyperplasia, inflammation or manipulation-related changes [3,19]. Although the markedly reduced free PSA ratio increased suspicion of clinically significant disease, neither parameter alone can reliably discriminate indolent from aggressive tumors.

Urinary molecular assays such as SelectMDx aim to refine risk stratification by evaluating gene expression patterns associated with high-grade prostate cancer. Nevertheless, molecular urine tests are not diagnostic tools per se; they provide probabilistic risk assessment and are influenced by pre-analytical variability, prostate volume and population characteristics [4,5,20]. Their sensitivity and specificity vary across cohorts, and independent validation studies have shown heterogeneity in predictive performance [21,22].

In this case, the test result indicated a high probability of clinically significant disease, which was later confirmed histopathologically.

However, it is important to emphasize that, given the markedly elevated PSA level and the presence of PI-RADS 4–5 lesions on mpMRI, the indication for prostate biopsy was already established. Therefore, the role of SelectMDx in this context was adjunctive rather than decision-defining, contributing to risk stratification rather than altering the diagnostic pathway [20,21].

From a practical perspective, the implementation of urinary molecular assays such as SelectMDx remains influenced by real-world factors, including accessibility, cost, and reimbursement policies. Availability may vary significantly across healthcare systems, and in certain regions, including parts of Eastern Europe, such tests are not routinely integrated into standard diagnostic algorithms. Consequently, their use should be considered complementary to, rather than a replacement for, established clinical and imaging-based decision-making [3,20].

The integration of mpMRI into the diagnostic pathway supports the detection of clinically significant prostate cancer and reduces unnecessary biopsies [6,7]. However, mpMRI interpretation remains operator-dependent and subject to interobserver variability, particularly in equivocal PI-RADS 3 lesions [23]. False-negative results may occur in small or anterior tumors, while false-positive findings may reflect prostatitis or post-biopsy artifacts.

Systemic inflammatory markers such as NLR have been proposed as prognostic indicators in urologic malignancies. Elevated NLR has been associated with adverse outcomes in both prostate and bladder cancer [9,10,11,24]. However, NLR lacks tumor specificity and may be influenced by infection, metabolic status and comorbidities. In the present case, NLR = 1.8 was consistent with organ-confined disease and absence of metastatic spread, yet no causal inference can be established.

An important consideration in biomarker-driven diagnostics is the potential risk of overdiagnosis and overtreatment. The increasing sensitivity of molecular assays and imaging modalities may lead to detection of low-volume or indolent tumors that might never become clinically relevant [25,26]. Although in this case the tumor was Grade Group 2 and therefore clinically significant, caution is warranted when generalizing biomarker-based intensification strategies. Multimodal biomarker integration may increase diagnostic sensitivity but also carries a risk of overdiagnosis and overinterpretation, particularly when multiple concordant findings are retrospectively aligned.

The synchronous detection of bladder carcinoma in situ following persistent hematuria underscores the importance of comprehensive evaluation beyond the initially suspected malignancy. CIS may present with minimal imaging abnormalities and requires high clinical suspicion [27,28]. The subsequent late hematuria episode in 2025 further highlights the complexity of surveillance in patients with orthotopic ileal neobladders. Intestinal epithelial shedding, chronic inflammation and reactive atypia may confound urinary cytology interpretation. Imaging modalities such as PET-CT provide valuable adjunctive reassurance when cytological findings are equivocal [29].

The apparent discrepancy between clinically detected synchronous cases and higher rates of incidental prostate cancer in cystoprostatectomy specimens further supports the concept that detection is strongly influenced by diagnostic pathways and clinical context [14,15].

Another clinically important aspect highlighted by this case is the role of diagnostic persistence when the clinical course does not fully align with the initially established diagnosis. In routine practice, persistent hematuria after prostate biopsy may easily be attributed to procedural trauma, transient mucosal irritation or post-interventional bleeding [19]. However, when bleeding persists beyond the expected interval or is accompanied by additional suspicious findings, a broader urothelial work-up becomes justified [16]. In the present case, further evaluation with uro-CT, urinary cytology and cystoscopy was essential for identifying a second primary malignancy that would not have been adequately explained by the prostate tumor alone. This emphasizes that multimodal diagnostics should not be viewed as a linear algorithm ending with the first confirmed diagnosis, but rather as a dynamic process that remains responsive to discordant clinical signals [17,18].

Emerging evidence suggests that systemic epigenetic alterations detectable in peripheral blood may reflect tumor-associated biological changes. Global DNA hydroxymethylation patterns in white blood cells have been proposed as exploratory biomarkers in prostate cancer, potentially complementing PSA-based stratification strategies [30]. Although not assessed in the present case, such systemic epigenetic markers illustrate the broader concept of host-derived biomarkers that may complement conventional imaging and molecular diagnostics.

Emerging molecular evidence suggests that synchronous urologic malignancies may be influenced by partially overlapping host-related, angiogenic and genomic pathways. Alterations involving tumor suppressor genes such as TP53 and RB1, dysregulation of cell-cycle and DNA repair mechanisms, as well as broader genomic instability patterns have been implicated in both prostate and urothelial carcinogenesis [31,32,33,34,35,36]. In parallel, recent studies have highlighted the growing role of urinary molecular biomarkers and transcriptomic profiling in improving risk stratification and characterization of aggressive urothelial and prostate malignancies [4,37,38]. Although these findings do not imply a common biological origin for synchronous tumors, they provide additional molecular context for interpreting complex multimodal diagnostic scenarios such as the present case.

Biomarker-based strategies aim not only to improve early detection but also to better characterize tumor biology and guide individualized clinical decision-making [4,37,38]. In prostate cancer, the combination of PSA-derived parameters, urinary molecular assays and multiparametric MRI has demonstrated improved diagnostic accuracy compared with single-modality approaches [4,17,18,34]. Similarly, advances in imaging and cytological evaluation have enhanced the detection of early bladder cancer lesions that may otherwise remain clinically silent [13,27].

In the present case, serum markers, urinary molecular testing, imaging, cytology and histopathology provided complementary information at different stages of the diagnostic pathway. However, this observation should not be interpreted as evidence of additive predictive value. Rather, it illustrates how multiple diagnostic modalities may be interpreted together in a complex clinical scenario when the initial diagnosis does not fully explain the subsequent clinical course.

The case also illustrates the practical value of integrating diagnostic modalities that reflect different dimensions of disease biology. Serum markers such as PSA provide systemic biochemical suspicion, urinary molecular assays contribute lesion-specific probabilistic stratification, imaging defines anatomical localization and extent, while histopathology remains the definitive diagnostic standard [20,21]. When these layers converge, the overall clinical interpretation may become more robust than when relying on a single diagnostic modality. At the same time, the later surveillance phase demonstrates that this principle remains valid after treatment as well: postoperative anatomy, inflammatory complications and equivocal cytology may again require combined interpretation rather than reliance on one modality alone [17,18]. This longitudinal diagnostic perspective represents one of the main strengths of the present report.

This report has inherent limitations. It represents a single case and does not allow extrapolation to broader populations. The apparent concordance between biomarkers may reflect cumulative testing rather than intrinsic biological correlation. Additionally, the association between low inflammatory status and favorable outcome should be interpreted cautiously.

Further limitations include the absence of quantitative correlation between biomarkers, imaging and histopathological findings, as well as the potential for selection bias inherent to case reporting. The integration of multiple diagnostic modalities also carries a risk of overinterpretation, particularly when concordant findings are retrospectively aligned.

Although the six-year follow-up is adequate to assess early and intermediate oncological outcomes, it may not fully capture very late recurrences or long-term functional complications.

Therefore, the observations presented in this case should be interpreted as descriptive and illustrative rather than predictive or generalizable.

Overall, this case should be interpreted as an illustrative example of clinical reasoning rather than evidence of diagnostic or therapeutic superiority.

## 4. Conclusions

This case does not introduce a novel diagnostic or therapeutic approach. Rather, it illustrates the importance of diagnostic persistence and multimodal correlation in a patient with synchronous prostate adenocarcinoma and bladder carcinoma in situ.

The findings should be interpreted strictly within the limits of a single case. No conclusions regarding additive predictive value, diagnostic superiority or broader clinical recommendation can be drawn from this observation. Nevertheless, the case highlights the practical relevance of continuing diagnostic evaluation when persistent clinical findings, such as hematuria, are not fully explained by the initial diagnosis.

## Figures and Tables

**Table 1:** Comparison of treatment strategies for synchronous prostate adenocarcinoma and bladder carcinoma in situ.

Strategy	Advantages	Limitations	Relevance to the Present Case
BCG + delayed prostate treatment	Bladder preservation	Requires strict adherence, risk of progression	Less suitable because of extensive multifocal CIS and need for prostate cancer treatment
Radical prostatectomy + BCG	Staged approach	Two treatments, delay risk	Possible staged approach, but may delay bladder CIS management
Radical cystoprostatectomy	Single-stage oncologic control	Higher morbidity, loss of bladder	Selected strategy due to simultaneous definitive treatment of both malignancies

**Table 2:** Long-term PSA dynamics and follow-up investigations.

Timepoint	PSA (ng/mL)	Clinical Events and Investigations
2019 (baseline)	17.69	Initial evaluation; elevated PSA, free PSA 6.5%
1-month post-op	0.008	Early postoperative PSA nadir
3-month post-op	0.008	Stable PSA
5-month post-op	0.008	Stable PSA
June 2020	0.06	CT thorax–abdomen–pelvis negative for recurrence; positive urine culture (*Klebsiella* spp.)
January 2021	0.034	No clinical and imaging evidence of recurrence
September 2021	<0.008	PSA undetectable
February 2022	0.011	Stable PSA
September 2022	0.008	Stable PSA
November 2023	<0.008	CT scan negative for recurrence
October 2024	0.008	CT scan negative for recurrence
October 2025	0.008	Newly detected microhematuria Urinary cytology: urothelial cells with nuclear atypia, intracytoplasmic vacuoles, coarse chromatin pattern, irregular nuclear membrane, metaplastic cells with severe dyskaryosis; numerous erythrocytes (intact and lysed). Cystoscopy no recurrence. CT scan: no oncological progression, stable findings
January 2026		PET-CT: metabolic activity at the level of the ileal neobladder could not be adequately assessed due to physiological tracer accumulation in urine; no FDG-avid lesions suggestive of distant metastases

Abbreviations: PSA—prostate-specific antigen; CT—computed tomography; PET-CT—positron emission tomography–computed tomography; FDG—fluorodeoxyglucose.

**Table 3:** Chronological diagnostic and therapeutic timeline of the case.

Year	Investigation	Result
2019	PSA	17.69 ng/mL
2019	Free PSA	6.56%
2019	NLR	1.8
2019	SelectMDx	90% PCa risk, 65% Gleason ≥ 7
2019	mpMRI	Two suspicious lesions (PI-RADS 4–5)
2019	Prostate biopsy	Adenocarcinoma Gleason 7 (3 + 4)
2019	Uro-CT	Bladder wall thickening
2019	Cystoscopy + biopsy	Multifocal high-grade CIS (pTis)
2019	Surgery	Radical cystoprostatectomy + ileal neobladder
2019–2021	Multiple positive urine cultures	Antibiotic treatment according to antibiogram
2025	PET-CT	No recurrence

Abbreviations: PSA—prostate-specific antigen; mpMRI—multiparametric magnetic resonance imaging; NLR—neutrophil-to-lymphocyte ratio; CIS—carcinoma in situ; PET-CT—positron emission tomography–computed tomography. The table summarizes the chronological sequence of diagnostic procedures, histopathological confirmation, surgical treatment and follow-up investigations in this case.

## Data Availability

The original data presented in this study are available on reasonable request from the corresponding author. The data are not publicly available due to privacy concerns.

## Abbreviations

PSA	Prostate-Specific Antigen
free PSA	Free Prostate-Specific Antigen
CIS	Carcinoma in Situ
PCa	Prostate Cancer
mpMRI	Multiparametric Magnetic Resonance Imaging
MRI	Magnetic Resonance Imaging
RT-PCR	Reverse Transcription Polymerase Chain Reaction
PI-RADS	Prostate Imaging Reporting and Data System
TRUS	Transrectal Ultrasound
CT	Computed Tomography
Uro-CT	Urographic Computed Tomography
PET-CT	Positron Emission Tomography–Computed Tomography
NLR	Neutrophil-to-Lymphocyte Ratio
ESR	Erythrocyte Sedimentation Rate
CRP	C-Reactive Protein
LUTS	Lower Urinary Tract Symptoms

## References

[1] Moschini M., Zaffuto E., Karakiewicz P.I., D’Andrea D., Foerster B., Abufaraj M., Soria F., Mattei A., Montorsi F., Briganti A. (2019). External Beam Radiotherapy Increases the Risk of Bladder Cancer When Compared with Radical Prostatectomy in Patients Affected by Prostate Cancer: A Population-based Analysis. Eur. Urol..

[2] Cumberbatch M.G.K., Noon A.P. (2019). Epidemiology, aetiology and screening of bladder cancer. Transl. Androl. Urol..

[3] Cornford P., van den Bergh R.C.N., Briers E., Van den Broeck T., Brunckhorst O., Darraugh J., Eberli D., De Meerleer G., De Santis M., Farolfi A. (2024). EAU-EANM-ESTRO-ESUR-ISUP-SIOG Guidelines on Prostate Cancer—2024 Update. Part I: Screening, Diagnosis, and Local Treatment with Curative Intent. Eur. Urol..

[4] Van Neste L., Hendriks R.J., Dijkstra S., Trooskens G., Cornel E.B., Jannink S.A., de Jong H., Hessels D., Smit F.P., Melchers W.J.G. (2016). Detection of high-grade prostate cancer using a urinary molecular biomarker-based risk score. Eur. Urol..

[5] Haese A., Trooskens G., Steyaert S., Hessels D., Brawer M., Vlaeminck-Guillem V., Ruffion A., Tilki D., Schalken J., Groskopf J. (2019). Multicenter optimization and validation of a 2-gene mRNA urine test for detection of clinically significant prostate cancer before biopsy. J. Urol..

[6] Kasivisvanathan V., Rannikko A.S., Borghi M., Panebianco V., Mynderse L.A., Vaarala M.H., Briganti A., Lars Budäus J., Hellawell G., Hindley R.G. (2018). MRI-targeted or standard biopsy for prostate-cancer diagnosis. N. Engl. J. Med..

[7] Ahmed H.U., El-Shater Bosaily A., Brown L.C., Gabe R., Kaplan R., Parmar M.K., Collaco-Moraes Y., Ward K., Hindley R.G., Freeman A. (2017). Diagnostic accuracy of multi-parametric MRI and TRUS biopsy in prostate cancer (PROMIS): A paired validating confirmatory study. Lancet.

[8] Rouvière O., Puech P., Renard-Penna R., Claudon M., Roy C., Mège-Lechevallier F., Decaussin-Petrucci M., Dubreuil-Chambardel M., Magaud L., Remontet L. (2019). Use of prostate systematic and targeted biopsy on the basis of multiparametric MRI in biopsy-naïve patients. Eur. Urol..

[9] Templeton A.J., Pezaro C., Omlin A., McNamara M.G., Leibowitz-Amit R., Vera-Badillo F.E., Attard G., de Bono J.S., Tannock I.F., Amir E. (2014). Simple prognostic score for metastatic castration-resistant prostate cancer with incorporation of neutrophil-to-lymphocyte ratio. Cancer.

[10] Marchioni M., Primiceri G., Ingrosso M., Filograna R., Castellan P., De Francesco P., Schips L., Cindolo L. (2016). The clinical use of the neutrophil to lymphocyte ratio (NLR) in urothelial cancer: A systematic review. Clin. Genitourin. Cancer.

[11] Li X., Ma X., Tang L., Wang B., Chen L., Zhang F., Zhang X. (2017). Prognostic value of neutrophil-to-lymphocyte ratio in urothelial carcinoma of the upper urinary tract and bladder: A systematic review and meta-analysis. Oncotarget.

[12] Ploussard G., Borgmann H., Briganti A., de Visschere P., Fütterer J.J., Gandaglia G., Heidegger I., Kretschmer A., Mathieu R., Ost P. (2019). Positive pre-biopsy MRI: Are systematic biopsies still useful in addition to targeted biopsies?. World J. Urol..

[13] Saginala K., Barsouk A., Aluru J.S., Rawla P., Padala S.A., Barsouk A. (2020). Epidemiology of Bladder Cancer. Med. Sci..

[14] Furrer M.A., Lyttwin B., Parli M.S., Burkhard F.C., Thalmann G.N., Wuethrich P.Y., Corcoran N.M. (2026). Incidental prostate cancer is frequently identified in men undergoing radical cystoprostatectomy for urothelial carcinoma of the bladder. Eur. Urol. Open Sci..

[15] Malte R., Kluth L.A., Kaushik D., Boorjian S.A., Abufaraj M., Foerster B., Rink M., Gust K., Roghmann F., Noldus J. (2017). Frequency and prognostic significance of incidental prostate cancer at radical cystoprostatectomy: Results from an international retrospective study. Eur. J. Surg. Oncol..

[16] Tan W.S., Feber A., Sarpong R., Khetrapal P., Rodney S., Jalil R., Mostafid H., Cresswell J., Hicks J., Rane A. (2018). Who should be investigated for haematuria? Results of a contemporary prospective study of 3556 patients. Eur. Urol..

[17] Kasivisvanathan V., Stabile A., Neves J.B., Giganti F., Valerio M., Shanmugabavan Y., Clement K.D., Sarkar D., Philippou Y., Thurtle D. (2019). Magnetic Resonance Imaging-targeted Biopsy Versus Systematic Biopsy in the Detection of Prostate Cancer: A Systematic Review and Meta-analysis. Eur. Urol..

[18] Woo S., Suh C.H., Kim S.Y., Cho J.Y., Kim S.H. (2017). Diagnostic performance of Prostate Imaging Reporting and Data System version 2 for detection of prostate cancer: A systematic review and diagnostic meta-analysis. Eur. Urol..

[19] Loeb S., Vellekoop A., Ahmed H.U., Catto J., Emberton M., Nam R., Rosario D.J., Scattoni V., Lotan Y. (2017). Systematic review of complications of prostate biopsy. Eur. Urol..

[20] Eyrich N.W., Morgan T.M., Tosoian J.J. (2021). Biomarkers for detection of clinically significant prostate cancer: Contemporary clinical data and future directions. Transl. Androl. Urol..

[21] Hendriks R.J., van Oort I.M., Schalken J.A. (2017). Blood- and urine-based biomarkers for prostate cancer: A review and comparison of novel biomarkers for detection and treatment decisions. Prostate Cancer Prostatic Dis..

[22] Wu H., Wu Y., He P., Liang J., Xu X., Ji C. (2024). A meta-analysis for the diagnostic accuracy of SelectMDx in prostate cancer. PLoS ONE.

[23] Rosenkrantz A.B., Verma S., Choyke P., Eberhardt S.C., Eggener S.E., Gaitonde K., Haider M.A., Margolis D.J.A., Marks L.S., Pinto P.A. (2016). Prostate Magnetic Resonance Imaging and Magnetic Resonance Imaging Targeted Biopsy in Patients with a Prior Negative Biopsy: A Consensus Statement by AUA and SAR. J. Urol..

[24] Gu X., Gao X., Li X., Qi X., Ma M., Qin S., Yu H., Sun S., Zhou D., Wang W. (2016). Prognostic significance of neutrophil-to-lymphocyte ratio in prostate cancer: Evidence from 16,266 patients. Sci. Rep..

[25] Eastham J.A., Auffenberg G.B., Barocas D.A., Chou R., Crispino T., Davis J.W., Eggener S., Horwitz E.M., Kane C.J., Kirkby E. (2022). Clinically Localized Prostate Cancer: AUA/ASTRO Guideline, Part I: Introduction, Risk Assessment, Staging, and Risk-Based Management. J. Urol..

[26] Welch H.G., Prorok P.C., O’Malley A.J., Kramer B.S. (2016). Breast-cancer tumor size, overdiagnosis, and mammography screening effectiveness. N. Engl. J. Med..

[27] Babjuk M., Burger M., Capoun O., Cohen D., Compérat E.M., Escrig D.J.L., Gontero P., Liedberg F., Masson-Lecomte A., Mostafid A.H. (2022). EAU Guidelines on Non–Muscle-Invasive Bladder Cancer 2022. Eur. Urol..

[28] Subiela J.D., Rodríguez Faba O., Guerrero-Ramos F., Palou J. (2020). Carcinoma in situ of the bladder: Why is it underdetected?. Curr. Opin. Urol..

[29] Fletcher J.W., Djulbegovic B., Soares H.P., Siegel B.A., Lowe V.J., Lyman G.H., Coleman R.E., Wahl R., Paschold J.C., Avril N. (2008). Recommendations on the use of 18F-FDG PET in oncology. J. Nucl. Med..

[30] Grelus A., Nica D.V., Miklos I., Belengeanu V., Ioiart I., Popescu C. (2017). Clinical significance of measuring global hydroxymethylation of white blood cell DNA in prostate cancer: Comparison to PSA in a pilot exploratory study. Int. J. Mol. Sci..

[31] Knowles M.A., Hurst C.D. (2015). Molecular biology of bladder cancer: New insights into pathogenesis and clinical diversity. Nat. Rev. Cancer.

[32] Attard G., Parker C., Eeles R.A., Schröder F., Tomlins S.A., Tannock I., Drake C.G., de Bono J.S. (2016). Prostate cancer. Lancet.

[33] Zhao M., Kim P., Mitra R., Zhao J., Zhao Z. (2016). TSGene 2.0: An updated literature-based knowledgebase for tumor suppressor genes. Nucleic Acids Res..

[34] Mjaess G., Chebel R., Karam A., Moussa I., Pretot D., Abi Tayeh G., Sarkis J., Semaan A., Peltier A., Aoun F. (2021). Prognostic role of neutrophil-to-lymphocyte ratio (NLR) in urological tumors: An umbrella review of evidence from systematic reviews and meta-analyses. Acta Oncol..

[35] Song Y., Hu J., Chen Q., Guo J., Zou Y., Zhang W., Chen X., Hu W., Huang P. (2018). Association between vascular endothelial growth factor rs699947 polymorphism and the risk of three major urologic neoplasms (bladder cancer, prostate cancer, and renal cell carcinoma): A meta-analysis involving 11,204 subjects. Gene.

[36] Peng Y., Song Y., Wang H. (2022). Systematic Elucidation of the Aneuploidy Landscape and Identification of Aneuploidy Driver Genes in Prostate Cancer. Front. Cell Dev. Biol..

[37] Song Y., Jin D., Ou N., Luo Z., Chen G., Chen J., Yang Y., Liu X. (2020). Gene Expression Profiles Identified Novel Urine Biomarkers for Diagnosis and Prognosis of High-Grade Bladder Urothelial Carcinoma. Front. Oncol..

[38] Peng Y., Dong S., Yang Z., Song Y., Ding J., Hou D., Wang L., Zhang Z., Li N., Wang H. (2021). Identification of docetaxel-related biomarkers for prostate cancer. Andrologia.

